# Systems-level insights into age-dependent innate immune responses in influenza B infection

**DOI:** 10.1128/spectrum.02656-25

**Published:** 2026-08-19

**Authors:** Shikha Saxena, Gurleen Kaur, Preeti Vishwakarma, Varun Kumar, Amit Kumar, Sonia Ompal, Satish Kumar, Sarjeet Singh, Akanksha Chauhan, Susmita Chaudhuri, Waseem Akram Malla, Sweety Samal

**Affiliations:** 1 Center for Virus Research, Therapeutics and Vaccines, Translational Health Science & Technology Institute, NCR Biotech Science Cluster 682813 , Faridabad, Haryana, India; 2 Manipal Academy of Higher Education 76793, Manipal, Karnataka, India; 3 Center for Microbial Research, Translational Health Science & Technology Institute, NCR Biotech Science Cluster 682813 , Faridabad, Haryana, India; 4 ICMR-National Institute of Malaria Research Field Unit, Guwahati, Assam, India; Barnard College, Columbia University, New York, New York, USA

**Keywords:** immune responses, influenza B, interferon, toll-like receptors, bulk RNA sequencing, aging

## Abstract

Aging is known to alter innate immune function, increasing susceptibility to viral infections, yet its specific effects on influenza B virus (IBV) infection remain poorly characterized. To address this gap, we developed a preclinical mouse model using a clinically relevant IBV strain Inf B/Phuket/3073/2013 to compare innate immune responses between young adult and aged mice, with a particular focus on age-related transcriptional and proteomic alterations in lung during infection. Bulk RNA sequencing and proteomics analysis of lung tissue harvested post-infection revealed distinct age-dependent transcriptional profiles. Bulk RNA-seq analysis identified 1,350 differentially expressed genes (DEGs), of which 824 exhibited an aged-biased expression pattern and were significantly upregulated in the lungs of influenza B virus-infected aged mice. Functional enrichment of these DEGs indicated perturbations in innate immune signaling, apoptosis, and pathways related to cellular stress and DNA damage in aged mice. There are several differentially expressed proteins that have been identified in young adult and aged influenza B infection. Among these differentially expressed proteins, some commonly co-expressed proteins have a role in virus entry, signaling pathways, and interferon response. Real-time PCR analysis confirmed altered expression of key innate immune genes, including Toll-like receptors (TLRs), interferon regulatory factors (IRF7 and IRF9), key antiviral genes (IFIT -2, IFIT-3 and STAT-1) and type I and II interferons (IFN-α, IFN-β, IFN-γ). Notably, young adult mice exhibited a more coordinated and robust activation of antiviral pathways, whereas aged mice demonstrated delayed and aberrant immune responses, characterized by excessive inflammation and impaired interferon signaling.

**IMPORTANCE:**

This study provides a comprehensive system-level analysis of age-driven alterations in innate immunity during influenza B virus (IBV) infection. While prior evidence has established that aging impairs antiviral responses across various viral infections, our findings specifically demonstrate that IBV infection in aged mice leads to a disproportionate and dysregulated transcriptional activation of innate immune pathways, including excessive type I interferon signaling and altered inflammatory mediator profiles, and uncover the potential therapeutic targets to improve health outcomes in the elderly population on influenza infection.

## INTRODUCTION

Influenza is a globally prevalent viral infection with significant clinical implications, associated with markedly higher rates of hospitalization and mortality in individuals over 65 years of age compared to younger adults (1, 2). Influenza B viruses (IBVs), which primarily infect humans (3, 4), are one of the two main types of influenza viruses responsible for annual seasonal epidemics of varying severity. IBVs have been shown to cause disease of comparable or, in some cases, greater severity than influenza A viruses (IAVs) (5), particularly contributing to a higher disease burden in children (6). During the 2020–2021 influenza season, the use of a quadrivalent vaccine was recommended in the United States to enhance protection against circulating IBV strains and remains the current recommendation (7). According to the weekly influenza surveillance report by the CDC, of the 993 influenza virus cases reported by public health laboratories, 88.21% were influenza A and 11.78% were influenza B (8). Populations at increased risk of IBV infection include children, the elderly, and immunocompromised individuals. Furthermore, there is a strong correlation between advancing age and increased morbidity and mortality following influenza virus infection (9, 10). A recent study on the protection and immunogenicity against influenza B, using a carbohydrate fatty acid ester adjuvant combined with a low-dose quadrivalent split-virion inactivated influenza vaccine, showed significantly lower hemagglutination inhibition (HI) titers against influenza B viruses compared to influenza A viruses (11). This disparity highlights a potential gap in the immune response elicited against influenza B, even with adjuvanted formulations. Understanding the immune mechanisms specific to the influenza B virus is, therefore, essential to optimize vaccine design and efficacy, particularly for vulnerable populations such as the elderly, who are at heightened risk of severe disease and poor vaccine responsiveness.

Aging is accompanied by significant alterations in both innate and adaptive immune function, a progressive decline collectively termed immunosenescence (12, 13). Aging affects all organ systems, including the immune system, suggesting that the age-related changes may directly influence the host response to the influenza virus. Spatial sequencing in influenza A-infected young adult and aged mice (scRNA and bulk RNA sequencing) has revealed a cell-type and age-dependent alteration in gene expression (14). In aged individuals, the decline in natural killer (NK) cell cytotoxicity contributes to impaired innate immune function. Additionally, dysregulated production of pro- and anti-inflammatory cytokines disrupts the activation and functionality of CD8^+^ T cells, thereby compromising efficient clearance of the influenza virus from pulmonary tissues (15, 16). Aged alveolar epithelial cells (AECs) have a higher frequency of senescence and secrete higher levels of the neutrophil-attracting chemokines CXCL1 and CXCL2 during influenza A infection (17). These findings highlight the importance of crosstalk among immune cells underlying the interaction between the host and the pathogen during influenza A infection. Influenza B viruses co-circulate globally with the influenza A virus and contribute significantly to seasonal influenza burden, particularly among children, the elderly, and high-risk groups (18–20). Unlike influenza A, influenza B evolves more slowly and has no animal reservoir, yet its clinical impact is often underestimated. Understanding age-related immune responses to influenza B infection is crucial for improving vaccine strategies and mitigating severe outcomes in vulnerable populations (21)

For the past few years, the influenza B virus strain Inf/B/Phuket/3073/2013 (Yamagata lineage) has been circulating widely among the population (22); however, the host–pathogen crosstalk during influenza B infection, particularly in the lung and across different organs *in vivo*, remains poorly understood. Understanding the immunopathogenesis of influenza B is critical for developing effective therapeutics and vaccines, especially as recurrent circulation of this strain poses a significant public health concern. In the *in vivo* studies, adaptation of influenza B (Yamagata lineage) viruses in mice and their propagation in eggs have been shown to enhance virulence, leading to increased lethality in mouse models (23). In a human epithelial cell line model, cells infected with influenza B virus (B/Brisbane/60/2008) were found to overexpress genes associated with immune response, autophagy, and apoptosis, providing useful insights into the dynamic interactions between the influenza B virus and epithelial cells (24). Despite reports suggesting reduced circulation, influenza B/Yamagata lineage viruses continue to be reported globally through the WHO FluNet surveillance platform (25).

The bulk RNA-seq provides a high-throughput approach to assess alterations in host-pathogen interaction, facilitating the discovery of novel pathways that could serve as potential therapeutic targets. Similarly, the proteomics approach can help uncover protein-protein interactions (PPIs) and virus-triggered alterations in host signaling pathways to identify critical host factors required for the virus infection (26, 27). There is a study on a preclinical animal model of influenza B virus infection (IBV strain of Victoria lineage [clade 1A]) in female BALB/c mice using an antigenically relevant IBV strain, for the anti-influenza drug testing and influenza-specific vaccine efficacy (28). In spite of the fact that mouse-adapted IBV strains derived from B/Phuket/3073/2013 and B/Florida/4/2006 have been reported in BALB/c mice, these experiments mostly centered on the adaptive properties and pathology of the viruses after repetitive passages in mouse lungs (23). To the best of our knowledge, however, there is no report describing the pathogenesis, susceptibility, and immune response to B/Phuket/3073/2013 in aged BALB/c mice. In this study, we developed a preclinical female BALB/c mouse model of infection with influenza B/Phuket/3073/2013 (Yamagata lineage) and applied a systems biology approach to investigate age-dependent host responses. To identify the host–pathogen interaction during influenza B virus infection, we employed an integrative approach by combining bulk RNA sequencing and mass spectrometry-based proteomics for age-associated transcriptional and protein level alterations in the lung of young adult and aged mice at 5 days post challenge. These aforementioned observations collectively suggest that the host-pathogen crosstalk in the lung is critical during influenza virus infection, and these interactions may be altered by aging. The improved understanding of these interactions might be critical for influenza virus infection. Our findings reveal that aging significantly alters the innate immune landscape following influenza B infection and highlight key gene and protein signatures that may serve as potential targets for future interventions against respiratory viruses and emerging viruses.

## MATERIALS AND METHODS

### Ethics and biosafety statement

The female BALB/c mice were bred in-house at the Experimental Animal Facility (EAF), THSTI. The young adult female BALB/c (6–8 weeks) and aged female BALB/c (>) were maintained in EAF (Experimental Animal Facility) at THSTI, Faridabad. The study was performed in compliance with CCSEA guidelines and approved by the THSTI’s Institutional Animal Ethics Committee (IAEC/THSTI#269) and the biosafety committee. All the mice were housed in single standard cages and kept under standard conditions of a 12 h light and 12 h dark cycle. All mice had free access to food and water *ad libitum*.

### BALB/c mice challenge model for influenza B and organ harvesting

The female BALB/c mice were used as a host for the influenza B/Phuket/3073/2013 virus. To establish the challenge model, the Inf/B/Phuket/3073/2013 virus was grown first in MDCK-L cells and then in 9- to 12-day-old embryonated chicken eggs, alternatively, and then the virus was lung passaged in female BALB/c mice. For lung passage, the female BALB/c mice (*n* = 3) were challenged intranasally (i.n.) with the virus, and the animals were observed for weight change up to 14 days. The lethal dose of Inf/B/Phuket/3073/2013 virus was calculated in both the young adult female BALB/c mice (*n* = 5) and aged female BALB/c mice (*n* = 5) (29). The growth kinetics was performed from the mouse-adapted (Inf B/Phuket/3073/2013) virus in MDCK-L cells. The 6-well plate of MDCK-L cells was infected with 0.1 MOI of mouse-adapted B/Phuket virus and the virus supernatant was collected at 6 h, 12 h, 24 h, 48 h, and 72 h post-infection. Then, the virus titer was estimated at all collected time points by the Reed and Muench method. In another experiment, the young adult and aged female BALB/c mice were divided into control and infected groups (*n* = 3). The animals in the infected group were challenged with 3 MLD_50_ of Inf B/Phuket/3073/2013 virus intranasally (i.n.) with 50 µL of virus administration by anesthesia induced with 4% isoflurane. Different organs (lung, spleen, kidney, brain, and liver) and Bronchoalveolar Lavage Fluid (BALF) were collected after the challenge of influenza at 3-, 5-, and 7-day post-challenge (dpc). For the organ collection, the mice were euthanized with 4% isoflurane, and the right ventricle was washed with 20 mL of cold PBS. The organs and BALF were collected in advanced MEM with 2× antibiotics. For the proteomics study, the lung was collected in liquid nitrogen.

### Determination of virus burden

The virus titer in different organs (lung, spleen, kidney, brain, and liver) and BALF was determined in MDCK-L cells. The MDCK-L cells were maintained in advanced MEM (Gibco) with 10% FBS and 1× penicillin-streptomycin at 37°C in 5% CO_2_. For the organ titer, MDCK-L cells were seeded in the 96-well plate, and a 10-fold serial dilution of the organ supernatant and BALF was prepared and overlayed on the cells for 1 h. The plates were incubated at 37°C in 5% CO_2_. After the incubation period, the plates were washed twice with PBS. The virus growth medium was added, and the plates were incubated further for 48–96 h for the observation of cytopathic effect (CPE). When the CPE was visible, the plates were washed, fixed in 4% paraformaldehyde, stained with 0.5% crystal violet, and then washed with distilled water. The CPE was quantified by the Reed and Muench method (30).

For quantification by real-time PCR, we infected young adult and aged female BALB/c mice (*n* = 9 each group with *n* = 3 animals at each time point) with 3 MLD_50_ of Inf B/Phuket/3073/2013 virus, with respect to mock-infected control female BALB/c mice (*n* = 9 each group with *n* = 3 animals at each time point) for both the age groups. The organs and BALF were collected after the challenge of influenza B virus at 3-, 5-, and 7-day post challenge (dpc) in TRI reagent (Invitrogen). The tissues were homogenized in TRIzol and then processed for RNA isolation. The cDNA was synthesized using Superscript III first-strand cDNA kit (Invitrogen) as per the manufacturer’s instructions. cDNA was checked by PCR with GAPDH primers. The list of primers used in the study is given in Table S1.

The viral copy number in the BALF and lung tissue at 3-, 5-, and 7-day post-challenge (dpc) was estimated by the matrix (M2e) specific gene of influenza by absolute quantification. For TLRs (TLR-1 to TLR-9) and IFNs (IFN type I and type II), relative quantification was done to estimate the fold change in the lung tissue at 5 dpc. GAPDH was used as an endogenous control gene. The fold change was calculated by the 2^−ddct^ method (31).

### Sample preparation for RNA-seq and analysis

The female BALB/c mice (young adult and aged, *n* = 3) were challenged with 3 MLD_50_ of influenza B/Phuket/3073/2013 virus as described above. The lungs were inflated, and small sections were collected in TRIzol at 5 dpc, kept overnight at 4°C, and then preserved at −80°C. The RNA was extracted using RNeasy Mini Kit (Qiagen, Cat# 74,104). The RNA samples were quantified and checked for purity and RNA integrity by nanodrop and qubit. For the library preparation, the NEB Ultra II directional RNA-Seq Library Preparation kit was used. The mRNA molecules were captured through magnetic Poly(T) beads, fragmented, and purified. The fragmented mRNA was reverse transcribed by the first-strand cDNA synthesis kit NEB Ultra II directional RNA-seq Library Prep kit (NEB, Cat# E7760L). RNA-seq data quality was assessed using FASTQC. Trimmomatic was employed in paired-end mode to filter reads, retaining only those with an average quality score of 25 within a 4-base sliding window. Reads shorter than 50 bases were subsequently removed. The high-quality reads were then aligned to the Mus musculus reference genome (GRCm39; v111) using HISAT2. For each sample, a separate GTF file was generated. These individual GTF files were merged into a single transcriptome using STRINGTIE. The merged transcriptome was indexed, and all samples were subsequently mapped to it for transcript quantification. Finally, the individual quantification files were imported into R and merged into a single count matrix using the “tximport” package. Initial quality control was performed using multidimensional scaling (MDS) on leading log-fold changes to evaluate sample clustering and identify biological outliers. Two outlier samples ("AG_CTR_3" and "AD_INF_1") were identified and excluded from further downstream analysis. The normalization factors were recalculated on the cleaned data set using the trimmed mean of M-values (TMM) method via calcNormFactors in edgeR. Low-expressed genes were independently filtered using the filterByExpr function. To protect against hyper-variable outlier genes, dispersion estimates and the QL model were fitted with the robust empirical Bayes method (estimateDisp and glmQLFit with robust = TRUE) while performing differential expression analysis. A log2 fold-change threshold of 0.58 (corresponding to a roughly 1.5-fold change) was applied using glmTreat. Genes were considered significantly differentially expressed (DEGs) if they exhibited a False Discovery Rate (FDR) adjusted *P*-value < 0.05.

To isolate the biological effects, various contrasts were constructed to evaluate (i) the baseline effect of aging in mock controls, (ii) the isolated viral response in adult hosts, (iii) the isolated viral response in aged hosts, (iv) the direct comparison of infected states, and (v) the synergistic interaction between aging and viral infection. The synergistic interaction was defined mathematically as the difference in viral response between the aged and adult cohorts using the following formula:


(Aged_Infected − Aged_Control) − (Adult_Infected − Adult_Control).

### Gene ontology and pathway enrichment analysis of differentially expressed genes by bulk RNA-seq

To interpret the functional consequences of the synergistic response, both Gene Set Enrichment Analysis (GSEA) and Over-Representation Analysis (ORA) were performed using the clusterProfiler suite. The DEGs from other contrasts were not subjected to this analysis. The Gene Set Enrichment Analysis (GSEA) was performed to identify co-ordinately regulated mechanistic pathways irrespective of arbitrary significance cutoffs; all genes from the interaction contrast were ranked using a composite metric incorporating both effect size and statistical significance as per following equation:


Score = sign (log2FC) x −log10 (P.value)

Ranked lists were tested against specific collections from the Molecular Signatures Database (MSigDB, via the msigdbr package for Mus musculus): (i) Transcription factor targets: MSigDB C3 collection, restricted to the TFT:GTRD subcollection. (ii) RNA stability and binding: MSigDB C5 (GO:BP) collection, strictly filtered for pathways matching "RNA_BINDING," "RNA_STABILITY," or "POST_TRANSCRIPTIONAL." (iii) General biological processes: Unrestricted MSigDB C5 (GO:BP) collection. Significance for GSEA was defined as an adjusted *P*-value < 0.05, with gene set sizes restricted to between 10 and 500 genes. The Over-Representation Analysis (ORA) was performed to differentiate between mechanisms of hyper-inflammation and functional suppression. Significant DEGs (FDR < 0.05) were analyzed separately based on their fold change. Directional ORA was then performed against the Gene Ontology (GO)-Biological Process using enrichGO, KEGG Pathways by using enrichKEGG (organism: “mmu”), and the Reactome Pathways by using the ReactomePA package (organism: “mouse”).

### Sample preparation for proteomics and analysis

The female BALB/c mice (young adult and aged, *n* = 3 for each control and infection) were challenged with 3 MLD_50_ of influenza B/Phuket/3073/2013 virus as described above. On 5 dpc, the lungs were collected from the infected and control female BALB/c mice and homogenized in RIPA lysis buffer (Himedia, #TCL131) containing protease inhibitor cocktail. The homogenized samples were centrifuged at 13,000 rpm for 15 min at 4°C. The supernatants were collected, and the protein was quantified using the BCA Protein assay (Thermofischer Scientific, #23225).

Infection in extracted lung samples was confirmed using Western blotting against nucleoprotein, and GAPDH was used as a loading control. The protein samples were resolved on 12% SDS-PAGE along with a pre-stained marker and transferred onto a polyvinylidene fluoride (PVDF) membrane. The membrane was blocked in 5% skimmed milk at room temperature for 1 h. The membrane was probed with anti-Nucleoprotein (1:1,000) (Abcam, #ab20711) and anti-GAPDH (1:1,000) (Thermo Fisher Scientific, #AM4300) antibodies at 4°C overnight. After washing with PBST, the blot was probed with anti-mouse HRP-conjugated secondary antibody (1:2,000) at room temperature for 1 h, and the membrane was washed thrice with PBST. The signal was developed using a femtoLUCENT PLUS-HRP G-Biosciences. The obtained blots were analyzed for obtaining the densities using the ImageJ software.

Furthermore, 500 μg of protein samples was precipitated using chilled acetone and incubated at −20°C. The samples were kept overnight at −80°C for further precipitation. The precipitated protein was pelleted at high speed for 15 min at 4°C. The supernatant was discarded, and the pellet was washed twice using acetone. The pellet, thus, obtained was further dissolved using 8 M urea. The dissolved protein was again quantified using the BCA Protein assay. One hundred micrograms of protein was used hereon for sample preparation. The samples were incubated for 1 h at 56°C after the addition of Dithiothreitol (DTT) (Merck, #3483-12-3), followed by the addition of Iodoacetamide (IAA) (Merck, I6125) with incubation for 1 h at room temperature. Protein samples were then digested with trypsin protease (Thermo Fisher Scientific, #90027) at 37°C for 18 h. The trypsinized samples were dried using a speed vacuum concentrator at 25°C, followed by desalting using Pierce C18 Zip-tips (Thermo Fisher Scientific, #87784) and again dried in a speed vacuum concentrator at 37°C. Each sample was run in duplicate on a Triple TOF 5600+ (SCIEX, Concord, Canada). Proteins were identified and quantified using MaxQuant software (v1.6, Max Planck Institute of Biochemistry, Germany). Statistical analysis was carried out using the Metaboanalyst web tool (v5). The groups were statistically analyzed using a two-sided unpaired Student *t*-test with a threshold *P*-value of 0.05. For pathway enrichment, over-representation analysis was performed with ShinyGO (v7.6) using the GO biologicals database (32). Metaboanalyst was used for principal component analysis and heatmap visualization.

### Validation of key genes identified in bulk RNA-seq data by real-time PCR

The eight key genes (innate immunity and age-related) (IFIT-2, IRF-7, IRF-9, STAT-1, ISG-15, Cdkn1a, IL1rn, and IFIT-1) were identified in bulk RNA-seq data and validated by real-time PCR using GAPDH as an endogenous control. A melt curve analysis was performed to determine the specificity of qRT-PCR. The relative expression of each sample was calculated using the 2^−ΔΔCt^ method with a control group calibrator (33). Two differentially expressed genes identified in bulk RNA-seq analysis (IFN-beta [1:1,000] [Abclonal #A22740] and ISG-15 [1:500] [Abclonal #A2416]) were validated by western blot. β-Actin (1:5,000) (Abclonal #AC004) was used as loading control. All the cDNA and protein samples used were the same as those used in bulk RNA-seq and proteomics analysis. All the samples were run in triplicate.

### Statistical analyses

The R package was used for the RNA-seq analysis. For the proteomics, statistical analysis was carried out using the Metaboanalyst web tool (v5). GraphPad Prism package 9.0 software was used for the statistical analysis of real-time PCR. Students’ unpaired *t*-tests and multiple *t*-tests were used for the RT-PCR and viral load analysis. Statistical significance was determined using the Holm-Sidak method, with alpha = 0.05. For direct comparisons between two groups, Student’s unpaired *t*-test was used, and for multiple comparisons between more than two groups, 1- or 2-way ANOVA was performed, followed by Tukey’s or Dunnett’s test to determine between-group differences. The differences were considered significant if *P* values were < 0.05. For RNA-Seq and pathway analyses, the FDR-corrected *P* value of 0.05 was taken as a threshold for significance.

## RESULTS

### Age-dependent host response model for influenza B virus

**Fig 1:**
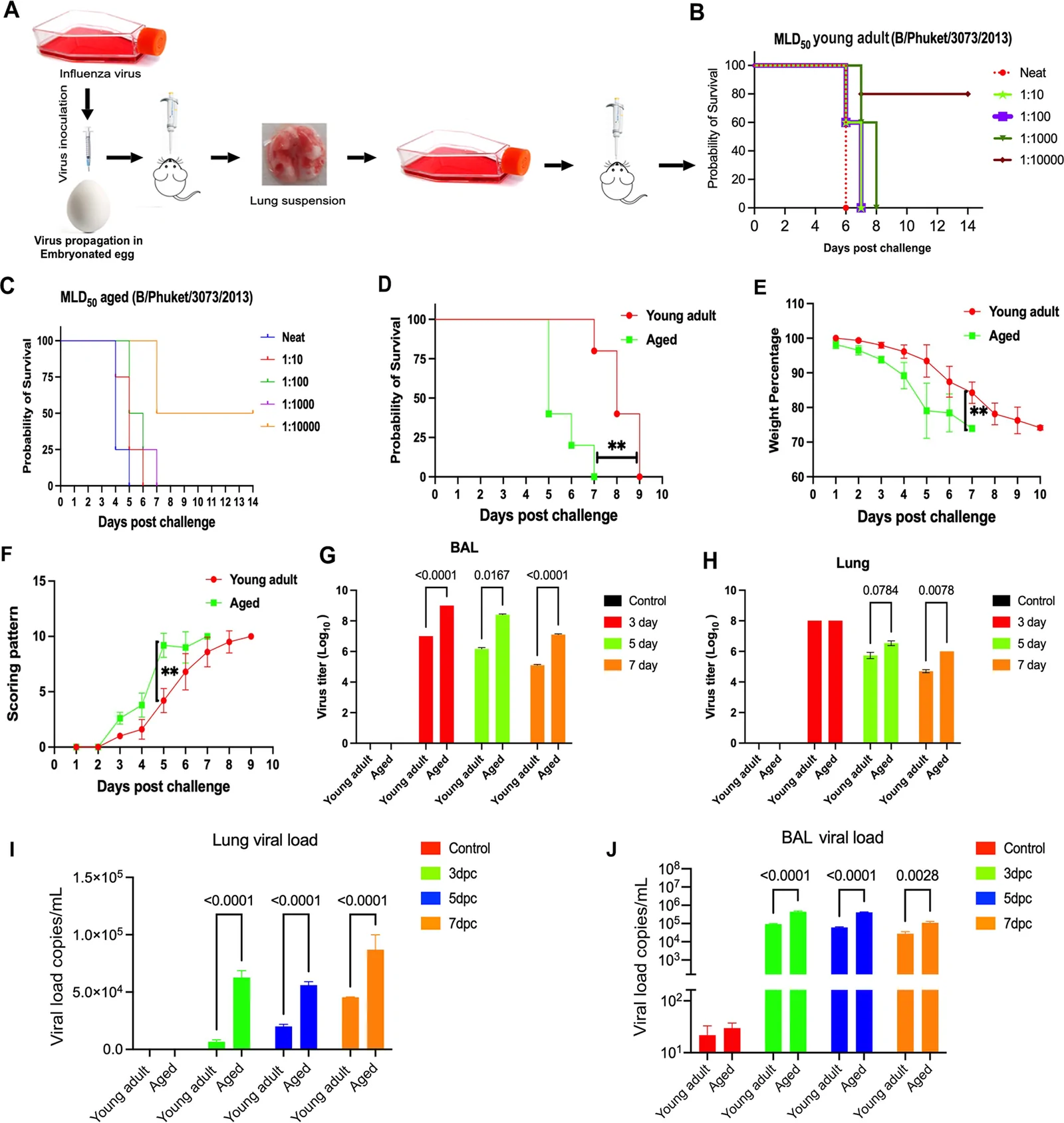
Lethality of influenza B (B/Phuket/3073/2013) virus (IBV) in young adult and aged female BALB/c mice. (**A**) Adaptation of influenza B (B/Phuket/3073/2013) in MDCK-L and embryonated eggs, then lung passages in young adult female BALB/c mice (*n* = 5) and (**B and C**) determination of MLD_50_ in female young adult and aged BALB/c mice (*n* = 5/group). (**D–F**) Survival, weight percentage, and scoring pattern in young adult and aged BALB/c mice (*n* = 5) after the 3 MLD_50_ of influenza B virus intranasal challenge (i.n.), The error bars indicate the standard errors of the mean. For statistical analysis, two-way ANOVA was performed. ****, *P* < 0.0001; ***, *P* < 0.001; **, *P* < 0.01; *, *P* < 0.05 compared to control groups. Determination of virus load and virus titer in B/Phuket/3073/2013 infected young adult and aged BALB/c mice (**G and H**). Virus titer in young adult and aged BALB/c mice infected with 3 MLD_50_ of B/Phuket/3073/2013 at 0 day, 3 days, 5 days, and 7 days post challenge in BAL and lung tissues in MDCK-L cells. Viral load of matrix (M2) gene of influenza in young adult and aged lung, and BALF (**I and J**). Viral load in young adult and aged lung, and BALF (*n* = 3) at 0 day, 3 days, 5 days, and 7 days post challenge (dpc) by real-time PCR. Data are mean ± SEMs from triplicate samples of a single experiment and are representative of results from three independent experiments and represent the *P* values by two-way ANOVA test.

Influenza viruses can infect most inbred mouse strains; however, certain strains are less susceptible and may require viral adaptation to cause overt disease. The recombinant influenza B/Phuket/3073/2013 infection in mice results in productive viral replication in the respiratory tract, accompanied by pulmonary inflammation and measurable clinical signs such as body weight loss. Studies have demonstrated that disease severity is associated with viral burden in the lungs and host inflammatory responses (34). Influenza B/Phuket/3073/2013 is generally susceptible to currently approved neuraminidase inhibitors, including oseltamivir (35). To establish a reliable and lethal challenge model for influenza B, we developed and validated a preclinical model using female BALB/c mice infected with the clinically relevant influenza B virus strain Inf B/Phuket/3073/2013 (Yamagata lineage) (Fig. 1A)**,** and its median lethal dose (MLD_50_) in both young adult and aged female BALB/c mice (Fig. 1B and C) was determined using standard protocols (30). We estimated the growth kinetics of mouse-adapted influenza B (Inf/B/Phuket/3073/2013) virus in MDCK-L cells at 6 h, 12 h, 24 h, 48 h, and 72 h post-infection. The virus titer was 10^2^, 10^3.67^, 10^4^, 10^6^, 10^7.33^ at respective time intervals (Fig. S1A). This strain demonstrated high virulence in female BALB/c mice. The virus exhibited high lethality and induced characteristic clinical symptoms in infected mice, including dullness, shivering, hunched posture, piloerection, and ruffled fur. A clinical scoring system was employed to quantify disease severity: Score 1—dullness with mild piloerection; Score 2—pronounced piloerection, reduced food intake, and shivering; Score 3—hunched posture and severe shivering.

Young adult and aged female BALB/c mice were challenged intranasally with 3 MLD₅₀ of the influenza B virus and monitored for 14 days post-infection. Survival rate, body weight changes, and clinical scores were recorded daily to assess the disease progression (Fig. 1D through F). Aged female BALB/c mice exhibited earlier onset and greater severity of disease following influenza B virus challenge compared to young adult mice. In the aged group, clinical signs—including piloerection, shivering, hunched posture, and reduced activity—appeared rapidly, with all animals succumbing to infection between days 5 and 7 post-challenge, resulting in 100% mortality. In contrast, young adult mice began to display signs of infection between days 7 and 9 post-challenge, also leading to 100% mortality by the end of the observation period. No mortality or clinical signs were observed in age-matched uninfected control groups. These findings demonstrate the successful establishment of a robust and reproducible preclinical mouse model for the influenza B virus. In addition, aged mice exhibited higher susceptibility to the influenza B virus-induced disease and earlier mortality, highlighting an age-associated difference in host response and disease progression.

### Elevated viral load and titer in the lungs and BALF of aged mice following influenza B infection compared to young adults

Aging plays a major role in innate and adaptive immunity. To investigate the impact of aging in mice on influenza B virus infection, different organs (lung, bronchioalveolar lavage fluid [BALF], kidney, spleen, brain, and liver) were collected on the 3rd day, 5th day, and 7th day post challenge (dpc) from young adult and aged mice. These organs were then processed for the virus titer determination by the Reed and Muench method. In young adult mice, the virus titer in the lung and BALF ranged from 10^7^ and 10^8^ TCID_50_/mL at 3 dpc, 10^6^ and 10^5^ TCID_50_/mL at 5 dpc, and 10^5^ and 10^4^ TCID_50_/mL at 7 dpc, respectively, whereas in the kidney, spleen, liver. and brain, no virus titer was observed in all three time points. In contrast, in the aged female BALB/c mice, the virus titer was observed in all the organs. In BALF, the titer was 10^9^, 10^8.5^, and 10^7^ TCID_50_/mL; in the lung, the titer was 10^8^, 10^6.5^, and 10^6^ TCID_50_/mL at 3 dpc, 5 dpc, and 7 dpc (Fig. 1G and H). In the spleen, the titer was 10^3^ TCID_50_/mL at 3 dpc, whereas at 5 dpc and 7 dpc, there were no virus titers observed. In the kidney at 3 dpc, the titer was 10^6^ TCID_50_/mL; at 5 dpc and 7 dpc, the titer was 10^1^ TCID_50_/mL. In the brain and liver, the titer was 10^4^ TCID_50_/mL at three dpc, whereas at 5 dpc and 7 dpc, no titer was observed (Fig. S1B).

Furthermore, the real-time PCR method was used to estimate the viral load in the lung and BALF. To do this, the lung and BALF from aged and young adult mice control vs infected groups were collected at 3rd dpc, 5th dpc, and 7th dpc. The viral load was estimated for the matrix gene (M2e) of influenza; a standard curve was generated for the absolute quantification in lung and BALF. In the lung, there were 8-fold, 2.5-fold, and 1.5-fold higher fold change in aged mice as compared to young adult mice, whereas in BALF, there were 4.8-fold, 7.5-fold, and 6.4-fold higher fold change at 3rd, 5th, and 7th days post challenge in aged mice as compared to young adult mice (Fig. 1I and J). These findings indicate increased susceptibility to influenza B virus infection in aged BALB/c mice relative to their young adult counterparts.

### Influenza B virus infection drives distinct transcriptomic signatures in young adult and aged mouse lungs

**Fig 2:**
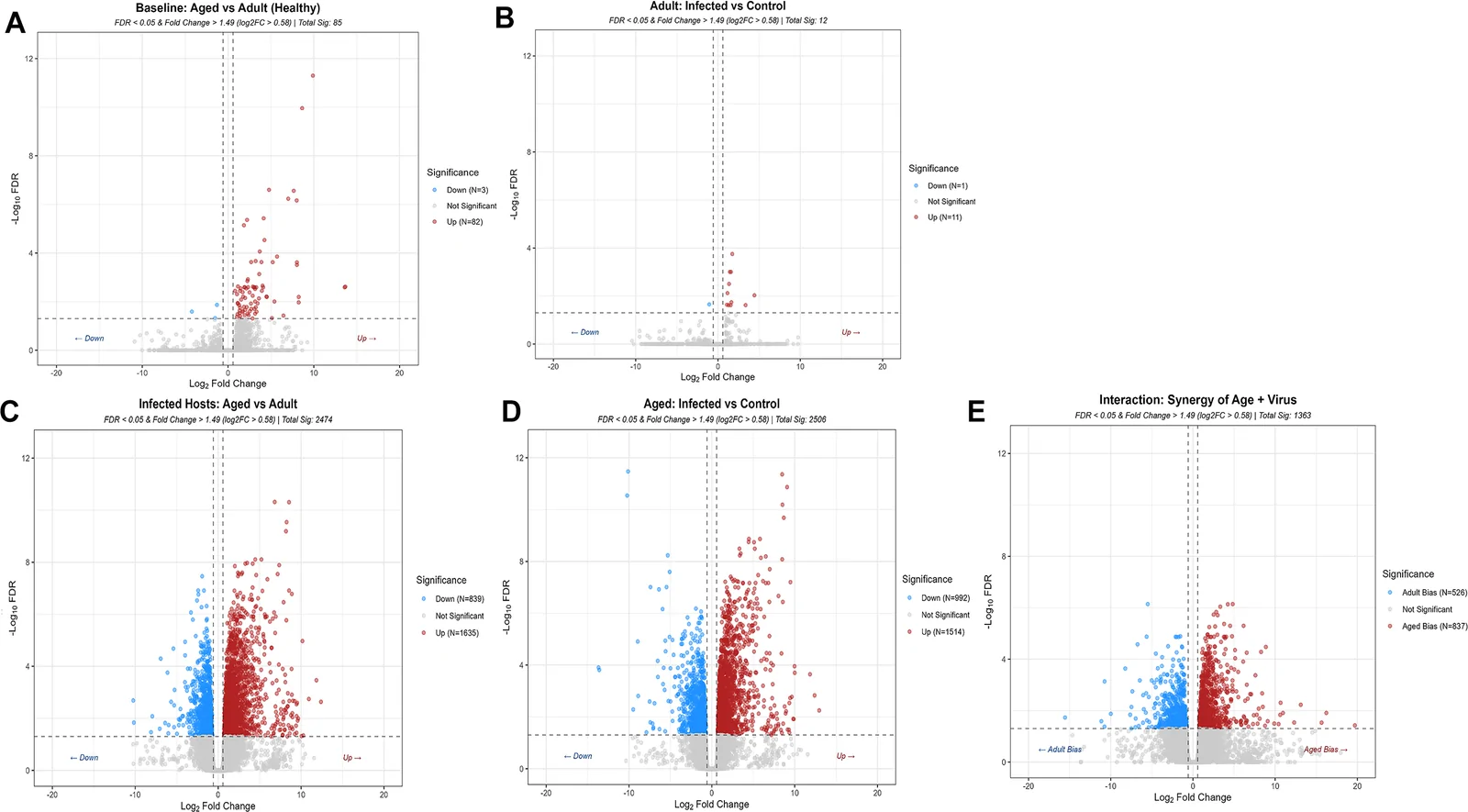
Volcano plots showing the differentially expressed genes (DEGs) in the lung of female young adult and aged BALB/c mice healthy vs influenza B (Inf B/Phuket/3073/2013) infection, in different comparisons at the FDR < 0.05 and log2FC > 0.58. (**A**) Young adult vs aged (healthy). (**B**) Young adult influenza B-infected vs young adult control. (**C**) Young aged-infected vs adult-infected. (**D**) Aged-infected vs aged control. (**E**) Interaction effect of age and infection status on differential gene expression.

Bulk RNA-seq profiling revealed a pattern of global transcriptional changes in the lungs of aged mice, marked by dysregulation of genes involved in inflammation, coagulation, and other immune-related pathways. To investigate age-associated transcriptional alterations in response to influenza B infection, we performed bulk RNA-seq on young adult and aged lung on day 5 post Inf B/Phuket/3073/2013 IBV infection. Previously, Angelidis et al. used a single-cell transcriptomic and deep proteomic atlas to study age-related responses in lung aging cells and reported increased transcriptional noise across ~30 cell types, especially marked cholesterol biosynthesis in type-2 pneumocytes and lipofibroblasts as key aging hallmarks (36). Our findings extend this understanding to influenza B, highlighting the importance of age-specific strategies to reduce severe outcomes in older individuals. In this study, we visualized the samples using PCA plots and observed that samples clustered by time and age, with the young adult mice infected with influenza B exhibiting more drastic changes in comparison to aged mice (Fig. S2). The volcano plots show that the baseline comparisons of uninfected aged vs adult lungs revealed a modest transcriptomic shift, with only 86 differentially expressed genes (DEGs) identified (83 upregulated, 3 downregulated; FDR < 0.05, |log2FC| > 0.58). Similarly, adult mice mounted a highly restricted response to the virus, yielding only 12 significant DEGs (11 upregulated, 1 downregulated). In contrast, viral infection in aged mice triggered a massive transcriptomic upheaval, resulting in 2,523 DEGs (1,521 upregulated, 1,002 downregulated) compared to age-matched controls. However, after controlling baseline differences due to age, we identified 1,350 DEGs (824 with an aged bias, i.e., upregulated in aged-infected lungs, 526 with an adult bias, i.e., downregulated in aged-infected lungs) (Fig. 2A through E). This indicates that the transcriptomic response in the aged lung is much more profound than merely an additive effect of baseline aging and viral infection.

Notably, several genes were found to be significantly upregulated in the lungs of aged mice. One such gene, *Oas1g*, is known for its role in the innate antiviral response and is also associated with cellular processes such as growth and apoptosis (37). Aging is associated with senescence, a cellular state marked by a lack of proliferation of the cells and resistance to apoptosis (38). Key age-associated genes such as *CSF3*, *Cdkn1a*, *OAS1*, *Ifnb1*, *IFI44L*, *Fcgr1*, and *IL1Rn* were found to be elevated in the aged group in comparison to young adults, whereas CSF3 and Cdkn1a genes were found to be expressed in the aged group only. In addition, upstream regulators of innate immune responses—including *SAA3*, *CXCL10*, *CCL7*, *ISG15*, *IFIT3*, *CCL12*, and *USP18*—also showed increased expression in the aged group in comparison to the young adult group.

These findings suggest a heightened and potentially dysregulated innate immune response in aged mice, which may contribute to the observed increase in disease severity and mortality and may be the ultimate result of age-mediated alterations in host-pathogen interactions in the lung following influenza B virus infection.

### Transcription factors and RNA-binding proteins as mechanistic drivers

To uncover the upstream regulators driving this dysregulated synergistic response, we performed targeted GSEA utilizing Transcription Factor (TF) and RNA-Binding Protein (RBP) gene sets. TF target analysis revealed that the synergistic transcriptomic profile is heavily regulated by targets of EGFR and MAPK3, pointing to the hyper-activation of mitogen-activated protein kinase signaling cascades. We also observed enrichment for targets of PSMB5 and PPARGC1A, linking the response to proteasomal degradation and metabolic reprogramming.

**Fig 3:**
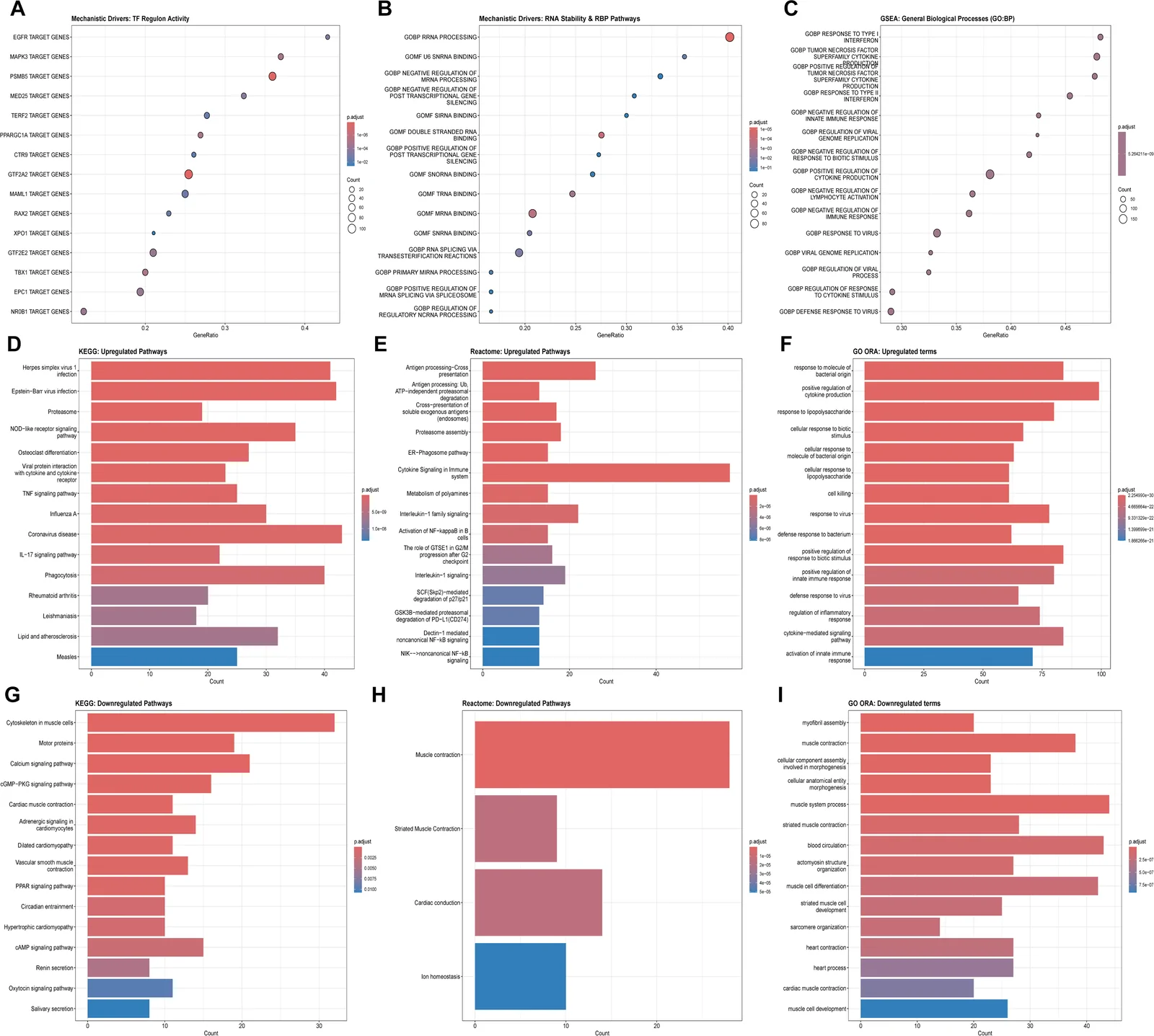
Functional enrichment analysis by dot plot and bar graph showing the significantly enriched biological pathways and regulatory networks identified from DEGs in aged BALB/c mice following influenza B virus infection. (**A**) Transcription factor (TF) target enrichment analysis, (**B**) RNA stability-associated pathways, (**C**) General Biological process pathways, (**D–F**) The Kyoto Encyclopedia of Genes and Genomes (KEGG), Reactome and Gene Ontology Over-Representation Analysis (GO-ORA) pathways for the top upregulated differentially expressed genes. (**G–I**) KEGG, GO-ORA pathways for the top downregulated differentially expressed genes. Only pathways with adjusted *P*-values of 0.001 are shown.

RNA stability and RBP pathways uncovered a massive dysregulation of post-transcriptional control mechanisms in the aged-infected lung. Highly enriched terms included “rRNA processing,” “U6 snRNA binding,” and “RNA splicing via transesterification reactions.” We also observed enrichment for the “negative regulation of mRNA processing” and “positive regulation of post-transcriptional gene silencing” (Fig. 3A through C). This indicates that the inflammatory response is not solely driven by transcriptional initiation but is heavily modulated by aberrant RNA splicing, stability, and post-transcriptional silencing machinery.

### RNA sequencing global kinetics and age-induced changes in young adult and aged mouse lungs

To understand the biological functions of the dysregulated genes, we performed Gene Ontology (GO), KEGG, and Reactome Over-Representation Analysis (ORA), alongside Gene Set Enrichment Analysis (GSEA). The pathways with an aged-bias (synergistically upregulated) were overwhelmingly dominated by hyper-inflammatory and hyper-reactive immune responses. GO:ORA revealed highly significant enrichment for the pathways such as “positive regulation of cytokine production,” “response to virus,” “defense response to bacterium,” and “activation of innate immune response.” The gene set enrichment analysis (GSEA) of general biological processes (GO:BP) confirmed widespread activation of interferon pathways “response to Type I interferon,” “response to Type II interferon,” and “tumor necrosis factor superfamily cytokine production.” The KEGG and Reactome pathway analyses further delineated these immune pathways. The upregulated genes in the KEGG analysis were heavily enriched for specific inflammatory signaling cascades, notably the “TNF signaling pathway,” “IL-17 signaling pathway,” “NOD-like receptor signaling,” and “Interleukin-1 family signaling.” Furthermore, the Reactome analysis highlighted the hyper-activation of “NF-kappa B in B cells” and intense “Proteasome assembly” and “Antigen processing (Cross-presentation)” mechanisms (Fig. 3D through F). Together, these data suggest that the aged lung reacts to the virus with an inflammatory response characteristic of a cytokine storm.

The over-representation analysis (ORA) of the 526 adult-biased (synergistically downregulated in the aged) genes revealed a severe suppression of structural and homeostatic tissue functions. GO:ORA of downregulated genes was exclusively populated by terms associated with muscular and structural integrity, including “myofibril assembly,” “muscle contraction,” “actomyosin structure organization,” and “blood circulation.” KEGG and Reactome analyses mirrored these findings, showing highly significant suppression of “Cytoskeleton in muscle cells,” “Vascular smooth muscle contraction,” “Calcium signaling,” “cGMP-PKG signaling pathway,” and general “Ion homeostasis” (Fig. 3G through I). The Reactome pathway gene networks of upregulated and downregulated genes in aged mice are shown in Fig. S3A and B. Altogether, these findings suggest that the infection leads to a severe loss of vascular tone, cytoskeletal breakdown, and impaired cardiopulmonary tissue function, which likely contributes to severe disease pathology in the aged mice. These observations further underscore the differential immune landscape in aged mice during influenza B virus infection.

### Identification of differentially expressed proteins in IBV infection among young adult and aged mice

**Fig 4:**
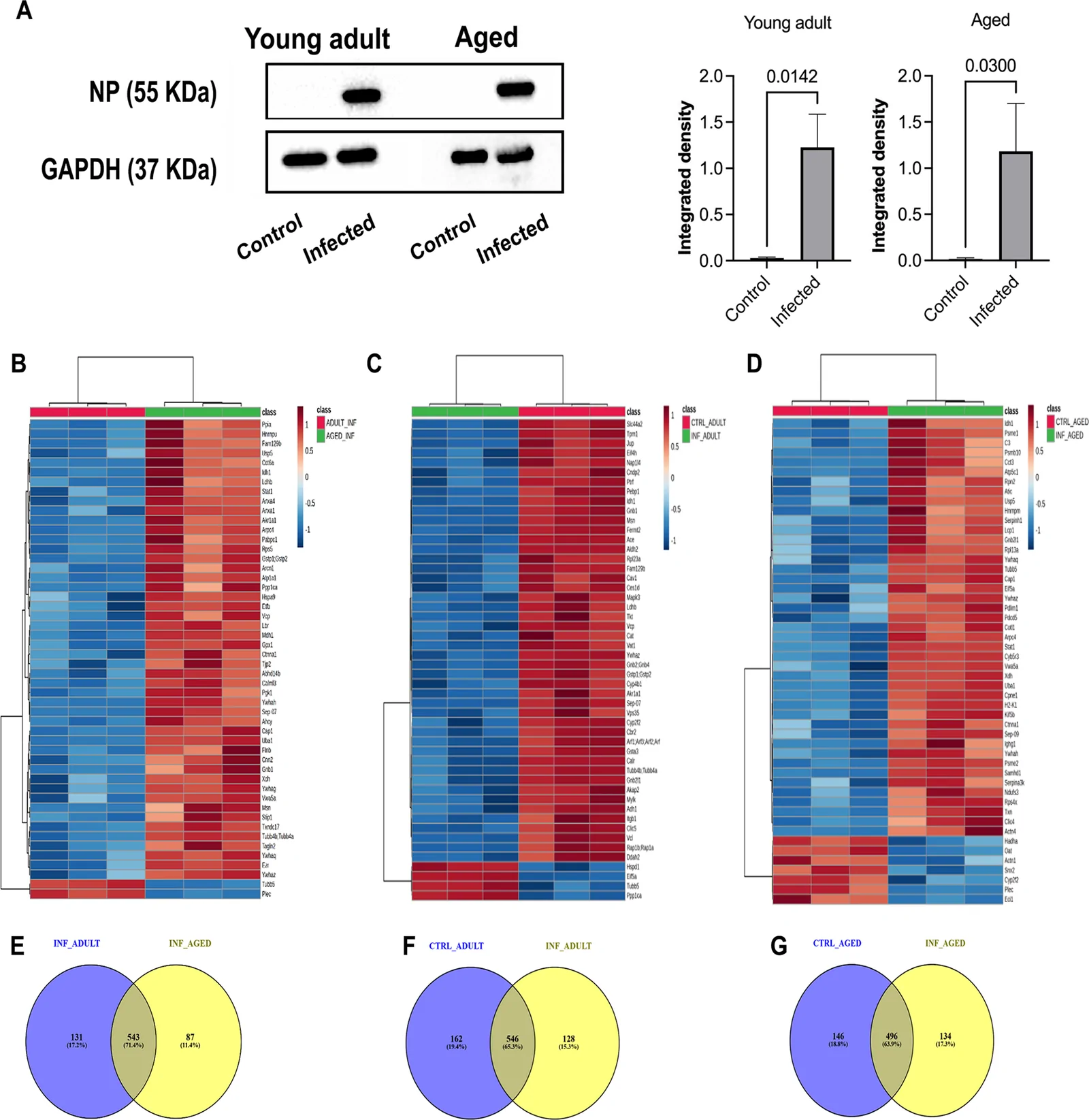
Identification of differentially expressed proteins in young adult and aged female BALB/c mice (*n* = 3) in influenza B (B/Phuket/3073/2013) infection. (**A**) The IBV infection in lung of young adult and aged BALB/c mice was assessed by IBV-specific NP antibody and the densitometry by western blot on 5 days post challenge. (**B–D**) The heatmap shows the hierarchical clustering of predicted top 50 differentially expressed proteins with FDR < 0.05 in three comparisons, IBV-infected young adult and IBV-infected aged mice, control young adult and IBV-infected young adult mice, and control aged vs IBV-infected aged mice. (**E–G**) Venn diagrams show the commonly co-expressed proteins across the three comparisons: IBV-infected young adult and IBV-infected aged mice; control young adult and IBV-infected young adult mice; and control aged vs IBV-infected aged mice. The groups were statistically analyzed using a two-sided unpaired Student *t*-test with a threshold *P*-value of 0.05.

Proteomic analysis offers a robust platform for the comprehensive identification of DEPs in virus infection. The identified DEPs are commonly involved in virus-host protein-protein interaction (PPIs) and regulate host signaling pathways, thereby uncovering key host factors essential for viral replication and propagation. In the study, we further performed the proteomics analysis in the lungs of young adult and aged female BALB/c mice upon influenza B infection at 5 dpc. To ensure the efficiency of virus infection before being subjected to mass spectroscopy analysis, viral NP proteins were visualized by Western blot in the lungs of young adult and aged mice following influenza B infection at 5 dpc. GAPDH was taken as an endogenous control gene. The western blot and its densitometric comparison in young adult and aged infection with NP-specific antibody were shown in Fig. 4A. A principal component analysis (PCA) of all identified proteins showed distinct clustering on IBV infection among young adult and aged mice, IBV-infected young adult and aged group, control young adult, IBV-infected young adult group, and control aged and IBV-infected aged group, healthy control young adult and aged group (Fig. S5A through C; Fig. 4C). The proteomics analysis listed a total of 761 DEPs that were identified in IBV-infected young adult and aged mice, in which 84 DEPs were upregulated and 4 DEPs were downregulated; whereas there was a total of 836 DEPs identified in control young adult vs IBV-infected young adult mice, in which 25 DEPs were upregulated and 203 DEPs were downregulated. A total of 776 DEPs were identified in control-aged versus IBV-infected aged mice, in which 95 DEPs were upregulated and 11 DEPs were downregulated.

A total of 578 DEPs were identified in control young adult vs aged mice, in which 19 DEPs were upregulated, and 104 DEPs were downregulated. The fold change was (log2FC ≥  2; FDR < 0.05). The heat map demonstrated the expression patterns of the DEPs; the heatmap was generated of the top variable DEPs in each comparison (Fig. S6A through C). The heatmap for the top 50 differentially expressed proteins was generated in each comparison (Fig. 4B through D; Fig. S4B). Among these differentially expressed proteins, some commonly co-expressed proteins have a role in virus entry, signaling pathways, and interferon response. The Venn diagrams illustrate the overlap of differentially expressed proteins (DEPs) identified in young adult and aged female BALB/c mice during influenza B virus (IBV) infection. A total of 543 DEPs were found to be commonly co-expressed across both age groups, irrespective of infection status. In the young adult, 546 DEPs were shared between the control and IBV-infected groups, whereas in the aged group, 496 DEPs were commonly expressed between control and infected mice (Fig. 4E through G). However, in the control adult and control aged, 772 DEPs were identified in which 578 DEPs were commonly expressed in both the groups (Fig. S4A).

These findings underscore the age-associated variation in proteomic responses to IBV infection and highlight a subset of DEPs that are consistently modulated during infection. Moreover, this analysis demonstrates the utility of quantitative proteomics in uncovering protein–protein interaction networks and regulatory nodes critical for host-pathogen interactions in the context of influenza B virus infection.

### Functional enrichment of differentially expressed proteins in the lungs of young adult and aged mice upon influenza B infection

**Fig 5:**
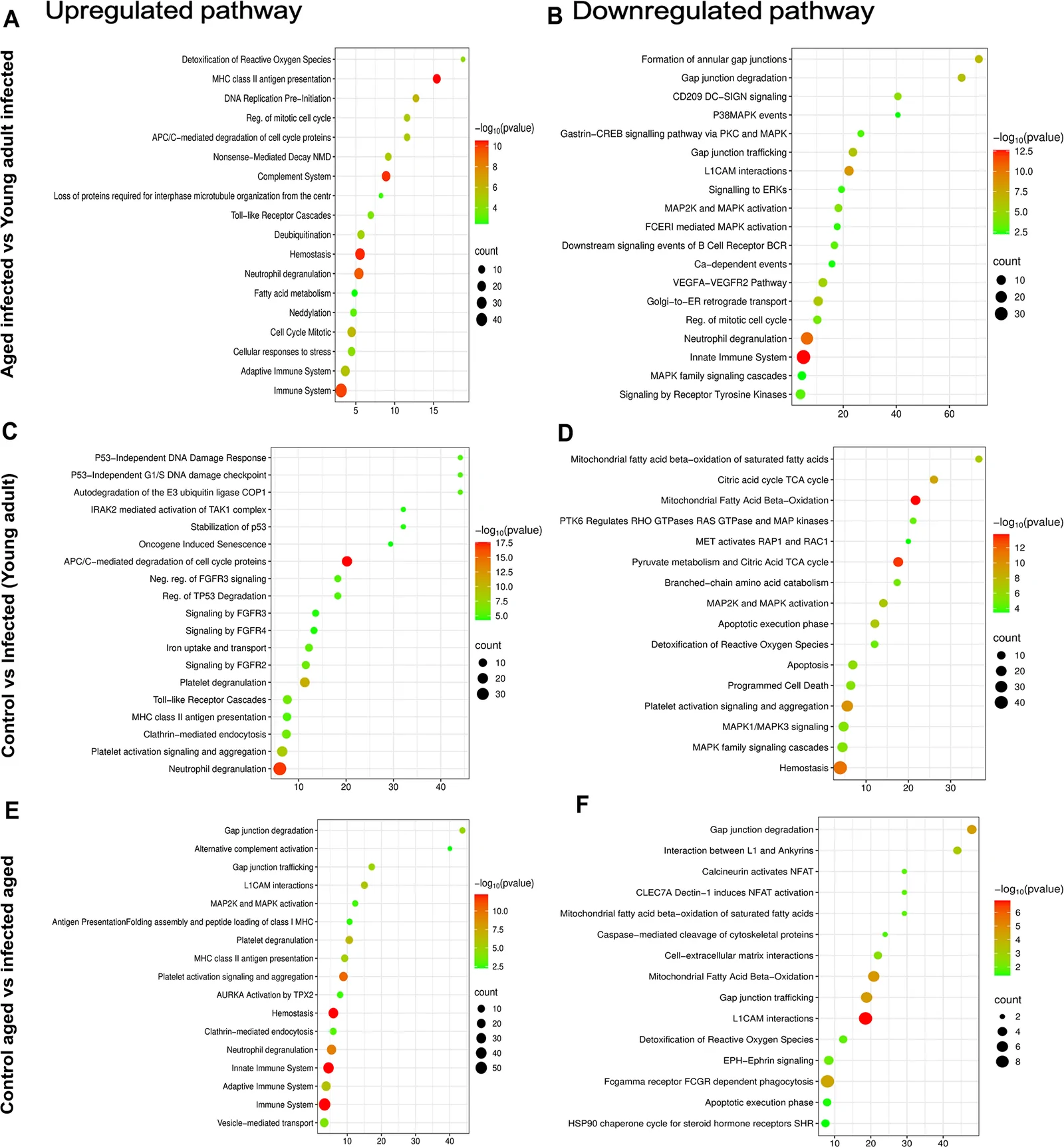
Functional categories of differentially expressed proteins, bubble diagram displays the Reactome pathway in lung of young adult and aged female BALB/c mice (*n* = 3) in influenza B (B/Phuket/3073/2013) infection. The differentially expressed proteins (DEPs) specifically identified in immune response, the upregulated and downregulated pathways in three comparisons, IBV-infected young adult and IBV-infected aged mice, control young adult and IBV-infected young adult mice, and control aged vs IBV-infected aged mice. (**A and B**) The upregulated and downregulated pathways in IBV-infected young adult and aged BALB/c mice. (**C and D**) Pathways in control young adult and IBV-infected young adult. (**E and F**) Pathways in control aged vs IBV-infected aged mice.

Functional enrichment analyses, including the Reactome pathway, were performed to investigate the roles of DEPs in the young adult and aged mice in IBV infection. In the bubble diagrams, the horizontal axis represents the enrichment factor, while the vertical axis denotes the functional pathways. The enriched DEPs were selected based on their association with immune response and aging. In the study, the Reactome pathway analysis was done for three comparisons for upregulated and downregulated pathways in the lung of aged vs young adult IBV-infected mice (comparison 1), IBV-infected adult mice vs control adult mice (comparison 2), IBV-infected aged mice vs control aged mice (comparison 3), and control adult vs control aged mice (comparison 4) (Fig. 5A through F and Fig. S4D and E). In comparison 1, MHC class II antigen presentation, toll-like receptor cascades, complement system, hemostasis, and other pathways were identified for upregulated differentially expressed proteins. For downregulated DEPs, MAPK family signaling cascades and MAP2K and MAPK activation pathways were seen to be downregulated. Additionally, the downstream signaling from the B-cell receptor, a crucial component of the adaptive immune response, was found to be downregulated. In comparison 2, gap junction degradation, alternative complement activation, and other pathways were identified in top upregulated DEPs, whereas in top downregulated DEPs, detoxification of reactive oxygen species, homeostasis, and MAP1/MAPK3 signaling and other pathways were identified. In comparison 3, there were MHC class II antigen presentation, negative regulators of FGFR3 signaling, toll-like receptor cascades, and other pathways that were identified in upregulated DEPs, whereas gap junction degradation, interaction between L1 and ankyrins were identified in downregulated pathways. Also, upregulation of p53-independent DNA damage response leads to chronic inflammation, thus leading to dysregulated immune homeostasis (39). Chronic activation of immune response pathways is associated with age-related immune dysfunction (40). In comparison 4, formation of gap junction, RHO GTPases activate CIT pathways was upregulated, whereas striated muscle contraction, apoptotic execution phase pathways were downregulated (Fig. S4E). Taken together, these identified pathways in the different comparisons may be essential in IBV infection and targetable for host-directed antiviral therapy.

### Influenza B virus induces distinct innate immune profiles in young adult vs aged BALB/c mice

**Fig 6:**
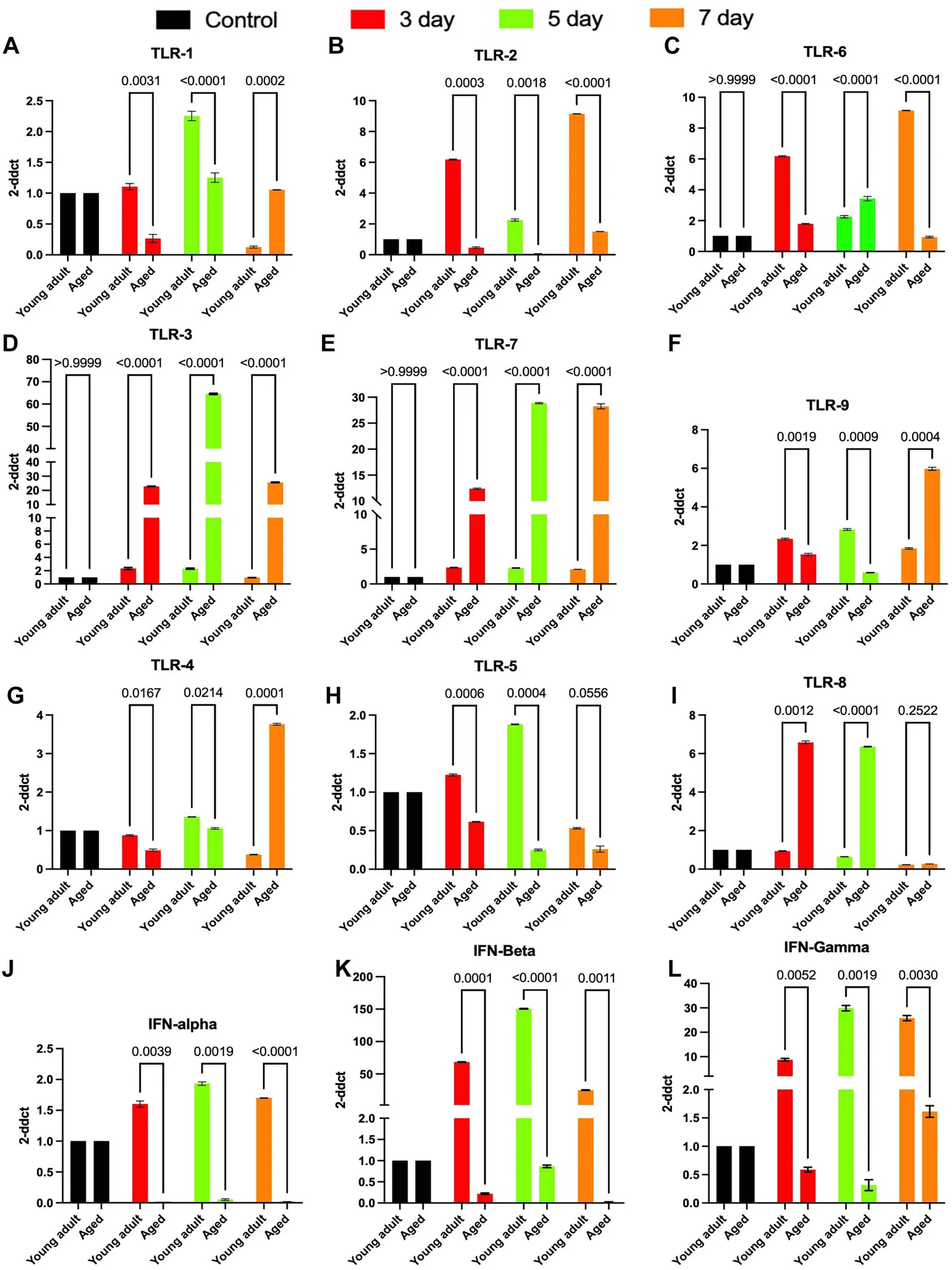
The relative mRNA expression of toll-like receptors (TLR 1–9) in the lungs of IBV (B/Phuket/3073/2013) infected young adult and aged female BALB/c mice (*n* = 3 for each time interval) at 0, 3, 5, and 7 days post challenge by real-time PCR, GAPDH was taken as an endogenous control in all the experiments. (**A–C**) Expression of TLR-1/2 and TLR-6 in young adult and aged BALB/c mice by real-time PCR at 0, 3, 5, and 7 days post challenge. (**D–F**) The relative expression of TLR-3, TLR-7, and TLR-9 in young adult and aged BALB/c mice. (**G–I**) The relative expression of TLR-4, TLR-5, and TLR-8 in young adult and aged BALB/c mice at 0, 3, 5, and 7 days post challenge. (**J–L**) The relative expression of interferon type I (IFN-α and -β) and type-II (IFN-γ) by real-time PCR in young adult and aged mice at 3, 5, and 7 days post challenge. The experiment was done with BALB/c (*n* = 3) mice for each time point and done in triplicate. Data are mean ± SEM (*n* = 3) with *P* values < 0.0001. The *P*-value summary (***P ≤ 0.001, **P ≤ 0.01, and *P ≤ 0.05, ns; not significant).

Next, we determined the expression of TLRs (TLR 1–9) in lung tissue of young adult and aged female BALB/c mice infected with influenza B/Phuket/3073/2013 virus on the 3rd, 5th, and 7th day post challenge by real-time PCR. The TLR 1/2, TLR 4, and TLR 2/6 are present on the cell surface and mediate the innate immune response. TLR-2 and TLR-6 mediate innate immunity in RSV-infected mice by promoting leukocytes’ production of inflammatory mediators (41). In the present study, TLR-1, TLR-2, and TLR-6 showed distinct age-dependent expression patterns. In young adult mice, TLR-1 was upregulated at 3- and 5-day post challenge but downregulated at 7-day post challenge. Whereas, aged mice showed the opposite trend, with downregulation at 3 and 5 dpi and upregulation at 7 dpi. TLR-2 was found to be upregulated across all time points in young adult mice but was downregulated throughout infection in aged mice. Similarly, TLR-6 was upregulated at 3 and 7 dpc and downregulated at 5 dpc in young adult mice, while aged mice displayed an inverse expression pattern, with downregulation at 3 and 7 dpc and upregulation at 5 dpc. These findings indicate a temporal and age-dependent dysregulation of TLR-mediated innate immune responses during influenza B infection, as shown in Fig. 6A through C.

TLRs-3, TLR-7, TLR-9, RIG-I, and MDA-5 are all known to respond to viral stimulation; their potential role in chronic airway inflammation is triggered by cytokine release (42). In this study, TLR3 and TLR7/8 were found to be downregulated in young adult female BALB/c mice compared to aged mice following influenza B virus (B/Phuket/3073/2013) infection. In contrast, TLR-9 expression was upregulated in young adult mice at 3 and 5 days post-challenge (dpc) relative to aged mice, but became downregulated by 7 dpc, suggesting a dynamic, age-dependent modulation of TLR-9 during IBV infection. The expression patterns of TLR-3, TLR-7, and TLR-9 in young adult and aged BALB/c mice following influenza B infection are presented in Fig. 6D through F. The expression of TLR-4 and TLR-5 following influenza B infection. TLR-5, a membrane-bound receptor that recognizes bacterial lipopolysaccharides and flagellin and also contributing to immunosenescence and dysregulated innate immune responses(43) . TLR-5 expression was elevated at 3, 5, and 7 days post-challenge (dpc) in young adult mice, whereas it was downregulated at all corresponding time points in aged mice. Similarly, TLR-4 expression was upregulated at 3, 5, and 7 dpc in young adult mice but downregulated in aged female BALB/c mice throughout the influenza B infection. Whereas the expression of TLR-8 was downregulated in young adults at 3 days and 5 days post challenge and upregulated in aged mice in the respective days, in the case of 7 days post challenge, the expression of this gene was not significant in both the groups (Fig. 6G through I).

We have further identified the expression of the IFN type I (⍺ and β) and type II (ɣ) in young adult and aged mice in influenza B infection. The type I and II IFNs were found to be upregulated in young adult mice, whereas downregulated in aged mice on influenza B virus infection (Fig. 6J through L).

### Validation of the key genes identified in bulk RNA-seq data among young adult and aged mice in influenza B infection

**Fig 7:**
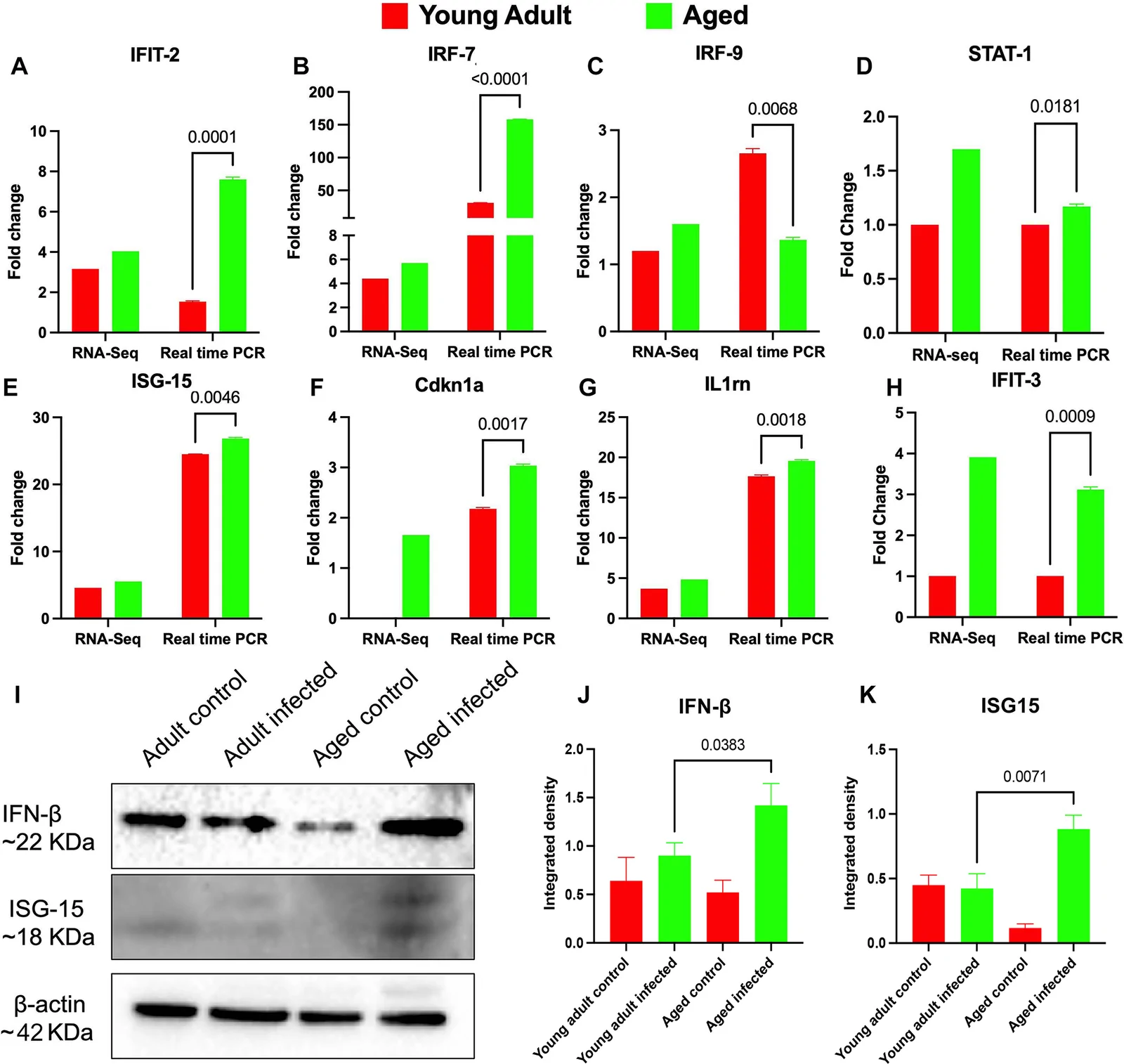
(**A–H**) Validation of identified immune response and age-related differentially expressed genes (IFIT-2, IRF-7, STAT-1, ISG-15, Cdkn1a, IL1rn and IFIT-3) by Real time PCR for the lung at 5 days post challenge in young adult and aged female BALB/c mice (*n* = 3) infected with B/Phuket/3073/2013 influenza virus. The expression patterns were compared with the bulk RNA-seq data to validate the identified DEGs. The samples used for the real-time PCR were the same replicates as used in bulk RNA-seq. In real-time PCR data, the GAPDH was taken as an endogenous control gene. (**I–K**) Validation of identified DEGs (ISG-15 and IFN-beta) by western blot in young adult and aged BALB/c mice following influenza B/Phuket/3073/2013 infection in the lung at 5 days post challenge. We have observed enhanced expression of IFN-beta and ISG-15 in aged infected mice as compared to adult infected also seen in integrated densitometry graphs in reference to β-actin. Data are presented as mean of three experiments ± SEM (*n* = 3) with *P* values < 0.0001. The *P*-value summary (***P ≤ 0.001, **P ≤ 0.01 and *P ≤ 0.05, ns, not significant).

The key genes that were commonly expressed and dysregulated during influenza infection in both adult and aged groups were further validated by quantitative real-time PCR (qRT-PCR) to confirm the transcriptomic findings (Fig. 7). Several genes involved in the innate immune response, including IFIT2, IRF7, IRF9, ISG15, STAT-1, and IFIT-3, were selected for validation by real-time PCR. These genes are known to play pivotal roles in antiviral defense—mediating interferon signaling, activator of transcription, viral RNA recognition, host anti-viral response, and restriction of viral replication. The qRT-PCR results demonstrated expression patterns consistent with those observed in the bulk RNA-seq analysis.

In addition, we examined Cdkn1a and IL1rn, two genes associated with the aging process and previously implicated in age-related modulation of immune responses during influenza virus infection (14). Cdkn1a is a cyclin-dependent kinase inhibitor linked to cellular senescence, while IL1rn encodes an interleukin-1 receptor antagonist that modulates inflammatory responses. In bulk RNA-Seq analysis, the expression of Cdkn1a was observed in aged lungs upon influenza B infection. In real-time PCR, the expression of this gene was found to be elevated in aged lung as compared to young adult lung. The qRT-PCR analysis confirmed that their expression changes were in concordance with the RNA-seq results (Fig. 7A through H). Moreover, we validated ISG-15 and IFN-β by western blot, identified as differentially expressed in bulk RNA-seq data, and in congruence with the transcriptomic data, IFN-β and ISG15 protein expression was found to be elevated in the aged mice following influenza B infection. Densitometric analysis of western blot bands revealed significant differences in IFN-β and ISG15 expression between young adult and aged mice. These findings corroborate the RNA-seq results and support the presence of age-dependent modulation of interferon-mediated antiviral responses during influenza B virus infection (Fig. 7I through K). These findings further support the age-dependent modulation of interferon-mediated antiviral responses during influenza B virus infection and highlighted the involvement of both innate immune mediators and aging-associated regulatory pathways in the host response across different age groups.

## DISCUSSION

In this study, we successfully established a lethal BALB/c mouse model for influenza B virus (IBV) infection using the clinically relevant B/Phuket/3073/2013 (Yamagata lineage) strain. This model faithfully recapitulates age-associated differences in disease progression, viral replication, and host immune responses, thereby serving as a valuable platform for studying influenza B pathogenesis and evaluating potential therapeutics or vaccine candidates.

Our findings demonstrate that aged BALB/c mice are markedly more susceptible to IBV infection than their young adult counterparts, as evidenced by earlier onset of clinical symptoms, higher clinical severity scores, and accelerated mortality. Notably, aged mice succumbed to infection between days 5 and 7 post-challenge, whereas young adult mice exhibited delayed symptom onset and reached 100% mortality between days 7 and 9. While these observations are derived from preclinical animal models, they align closely with human epidemiological data, highlighting the increased susceptibility of the elderly to severe outcomes following influenza infection (44). Quantification of viral titres and viral load revealed significantly higher levels of virus in the lungs and bronchoalveolar lavage fluid (BALF) of aged mice. Moreover, aged mice exhibited systemic viral dissemination with detectable titers in extrapulmonary organs such as the kidney, liver, spleen, and brain, particularly at early time points. These results suggest impaired viral clearance and a compromised barrier function in aged hosts, consistent with immunosenescence and reduced innate antiviral efficacy. Similar to our findings, Dwivedi et al. reported enhanced viral susceptibility in aged K18-hACE2 transgenic mice during SARS-CoV-2 infection, underscoring the impact of aging on disease severity (45, 46). The influence of host age on Chikungunya encephalitis has been highlighted by Anderson et al., who reported age-dependent differences in disease outcome (45)

Although this study did not investigate the mutations acquired during adaptation that may contribute to the enhanced viral replication and increased lethality of the adapted strain in young adult mice compared with the parental virus, several other findings support the suitability of this challenge strain for studying age-dependent innate immune responses. In particular, the strain consistently induced distinct disease outcomes and host responses in adult and aged mice, making it a valuable model for investigating the mechanisms underlying age-associated differences in influenza pathogenesis.

Bulk RNA-seq profiling further revealed profound age-dependent differences in host transcriptomic responses to IBV. While young adult mice showed moderate transcriptional alterations, aged mice exhibited a highly dysregulated gene expression profile, with more than 1,300 differentially expressed genes, including key players in inflammation, antiviral defense, and coagulation pathways. Several DEGs involved in innate immune regulation processes, including Oas1g, Trim30d, Irf7, Ifi20b, Isg15, Usp18, Trex1, Dhx58, and Stat2, were found to be elevated in aged lung tissue. Oas1g functions as a major ISG in Alzheimer’s disease, potentially downregulating the activation of IFN-β and STAT1 and reducing the expression of ISGs involved in neuroinflammation (47). In our study, this gene was found to be associated with most of the immune system-related pathways. Genes associated with T-cell-mediated immunity, such as Ifnb1, Rsad2, Gm49339, Trex1, and Gzmb, were also upregulated. Additionally, DEGs related to inflammatory responses, including Saa3, Cxcl10, Cxcl9, Ccl2, and IL1rn, showed increased expression in aged female BALB/c mice lungs. Furthermore, key antiviral innate immune response genes like Ifit3, Usp18, and Dhx58 were found to be upregulated in the aged lung of female BALB/c mice. Collectively, the pathway analyses suggest that the aged mice are more prone to homeostasis, apoptosis, inflammation, and thrombosis following influenza B infection as compared to young adult mice. Several innate immune genes (e.g., Oas1g, ISG15, CXCL10, CCL7, USP18) and age-associated genes (Cdkn1a, Csf3, Ifnb1) were significantly upregulated in aged mice, suggesting a heightened, but likely dysregulated, innate immune response that may contribute to immunopathology. Cdkn1a, an age-related gene, associated with the senescence marker of p21 (48). In a recent study on influenza A, scRNA-seq data from the lungs of aged mice expressed significantly higher levels of Cdkn1a than did those of young mice pooled from different timepoints (14). The age-related transcriptional signatures further highlight the importance of considering host age in the design of therapeutic strategies and in understanding influenza B pathogenesis. In our study, this gene was found to be elevated in aged lung upon influenza B infection; we have validated this gene with real-time PCR.

Aged mammary glands exhibit signs of pro-inflammatory microenvironment, with increased production of inflammatory cytokines like colony-stimulating factor CSF3 supplementation/receptor (CSF3R) and IL6 (49). We also found a higher expression of the Cdkn1a gene in the IBV-infected mice’s lung compared to the young adult mice. Fcgr1 gene, a known target of IFNG (50) , was upregulated in the aged lung compared to the young adult lung of female BALB/c mice. IL1RN gene inhibits the activities of interleukin 1, interleukin 1 alpha, and interleukin 1 beta and modulates the immune and inflammatory responses. It was found to be upregulated in the aged lung of female BALB/c mice. Tnfrsf9, a member of the TNF receptor family, is a pro-inflammatory cytokine that was dysregulated by aging (51) . TNF is a well-characterized inflammatory cytokine with critical roles in regulating cell proliferation, apoptosis, and morphogenesis. It is also implicated in the pathogenesis of numerous human diseases (52) .

IFN-induced protein 44-like (IFI44L) is a paralog gene of IFI44. IFI44L is an interferon-stimulated gene (ISGs), induced by many different viruses such as IAV and respiratory syncytial virus (53)(54) . Overexpression of IFI44L has been associated wit the persistence of hepatitis E virus infection (55). Guanylate-binding protein 3 (GBP3) is an interferon-inducible GTPase vital for cell-intrinsic immunity and inflammasome activation. This gene promotes the pathogen sensing and restricts the viral infection upon activation (56). Functional enrichment analyses revealed age-associated changes in the host response to influenza B virus infection. Aged mice exhibited marked enrichment of inflammatory and antiviral pathways, including cytokine production, interferon signaling, TNF, IL-17, NOD-like receptor, and NF-κB signaling, whereas genes associated with tissue integrity, cytoskeletal organization, muscle contraction, vascular function, calcium signaling, and ion homeostasis were significantly suppressed in aged mice (57, 58). Transcription factor target analysis showed that the observed transcriptomic response was strongly associated with genes regulated by EGFR and MAPK3 in influenza B infection. MAPK signaling plays a critical role in regulating inflammatory responses. Enrichment of PSMB5 target genes points to enhanced involvement of the ubiquitin–proteasome system. The enrichment of PPARGC1A-associated targets further suggests alterations in cellular metabolic pathways, indicating that influenza B virus infection may induce metabolic reprogramming. Altogether, EGFR/MAPK signaling, proteasomal activity, and metabolic pathways may contribute to the dysregulated host response following the influenza B infection (59, 60).

Notably, the homeostasis-related pathways were also enriched in the aged lung in IBV infection. The dysregulation of age-related homeostatic genes involves the proliferation of CD4 and CD8 T-cell subsets, including Tregs, in response to influenza vaccination in the elderly population (61). These outcomes might be associated with impaired cellular functions and age-mediated alterations in immune-endothelial and epithelial interactions in the lung following respiratory virus infections. Proteomic analyses corroborated the transcriptomic findings, identifying several differentially expressed proteins (DEPs) with known roles in viral replication and immune regulation. Proteins such as IFI35, IRGM1, DDX3X, and ITGB1 were elevated in aged lungs and have been previously implicated in interferon signaling, viral sensing, and immune evasion (62, 63). HNRNPUL2, also known as (hnRNP A2/B1), acts as a pro-viral factor in IAV infection, found to be enriched in aged lungs following IBV infection. HnRNP A2/B1 is an interacting partner of the influenza viral protein NS1, and it is found that it suppresses NS1 RNA/protein levels and NS1 mRNA nucleocytoplasmic export in influenza A virus infection (64). The Mapk3 gene is also found to be enriched in aged lungs. It is reported that MAPK13-IN-1 has antiviral activity against SARS-CoV-2 (65). In IAV infection, identification of 16 proteins targeting host factors suppresses IAV replication, with two targeting CDK2 and FLT3 showing pan-antiviral activity across influenza and coronavirus families (66). There are some key host proteins and kinases that have been identified in IAVs (H1N1, H3N2, and H5N1) and host-directed therapeutic targets such as AHNAK and COPB1, which showed pan-antiviral activity against both IAV and SARS-CoV-2 (67). Collectively, these findings represent the novel potential of identified differentially expressed proteins in influenza B infection. Although we have identified several DEPs and DEGs in transcriptomics as well as the proteomics data, the limitation of this study is that transcriptomic and proteomic analyses were performed at a single time point (5 dpc), which was selected to capture the pronounced age-dependent differences in disease severity. The temporal dynamics of host responses throughout infection could not be assessed at a single time point, and future longitudinal multi-omics studies are warranted to better define the age-associated immune responses to influenza B virus infection. Nevertheless, the consistent identification of these DEPs in proteomic data sets underscores their potential importance in age-dependent IBV pathogenesis.

Furthermore, targeted analysis of pattern recognition receptors and interferons revealed downregulation of TLRs (particularly TLR-1, TLR-2, and TLR-6) and type I/II interferons in aged mice, contrasting with their upregulation in young adult mice. TLR-3 recognizes dsRNA (41) and a molecular signature for most of the viruses, TLR-7/8 recognizes ssRNA (68), and TLR-9 recognizes unmethylated CpG motifs in viral DNA (69); these TLRs activate the MyD88-dependent pathway. In our study, these TLRs are found to be downregulated in young adult lung and upregulated in aged lung. Age-related altered TLR responses may contribute to the risk of infectious disease in the older population. There is a study on age-dependent TLR function in human macrophages during West Nile virus infection, in which elevated TLR-3 levels increase cytokine levels and suggest a possible mechanism for the increased severity of WNV infection in older individuals (70). Overall, the temporal variation in TLR expression observed in aged mice is consistent with previous reports showing that aging alters the kinetics of innate immune signaling, resulting in delayed or dysregulated activation of TLR pathways during influenza infection, rather than sustained regulation across all time points (60, 71). It is reported that Geriatric PBMCs have significantly less interferon alpha in response to influenza virus infection as compared to younger controls. This could have a deleterious effect on anti-influenza responses in the geriatric population and contribute significantly to the susceptibility of older individuals to influenza (72). Type I and type II interferons play crucial roles in the immune response to viral infections; their function can be compromised with age, contributing to higher susceptibility and severity of viral diseases in older young adults (73). This may suggest a blunted early innate immune response in aged hosts, possibly contributing to delayed viral recognition and exacerbated disease. IFI35 (interferon-induced protein 35) inhibits swine (H3N2) influenza virus replication by directly interacting with the viral protein NS1 and regulates RIG-I-mediated antiviral immunity (74). Irgm1 (immunity-related GTPase M1) is a negative regulator of IFN-I response in cGAS-STING and RIG-I-MAVS pathways (75). ITGA3 is an integrin alpha 3 gene, which is among the top genes that upregulate the HIV-1 virus replication (76). Ddx3x, Hnrnpu, and Itgb1 genes are also involved in host defense mechanisms in influenza virus, playing a pivotal role in innate immunity by facilitating chromatin opening to activate immune genes during host defense and promoting peripheral entry by Rabies Virus (77, 78) . In our study, these proteins were found to be elevated in the lung following IBV infection in comparison to young adult mice. This study has several limitations that should be acknowledged. First, transcriptomic analyses were performed on whole-lung tissues, which may mask cell-type-specific transcriptional changes and limit the resolution of cellular responses contributing to disease pathogenesis. Second, the pathway enrichment analyses presented here are primarily descriptive and do not establish causal relationships between the identified pathways and disease outcomes. Functional validation of key molecular pathways and regulatory networks will, therefore, be required to confirm their biological significance, future longitudinal multi-omics analyses across early and late stages of infection in larger cohorts.

### Conclusion

Collectively, our findings provide important insights into the age-dependent host responses that shape disease outcomes following influenza B virus infection. By integrating virological, pathological, transcriptomic, and proteomic analyses, we demonstrate that aging is associated with distinct immune and inflammatory signatures that contribute to enhanced disease severity. Importantly, the aged mouse model described here recapitulates key features of severe influenza observed in elderly populations and represents a suitable preclinical platform for investigating influenza B virus pathogenesis, evaluating candidate therapeutics, and advancing the development of age-specific vaccine strategies. The molecular pathways and host-response signatures identified in this study offer a valuable foundation for future mechanistic investigations and may facilitate the discovery of biomarkers and therapeutic targets associated with severe disease in aging populations. Although the circulation of the influenza B/Yamagata lineage has markedly declined in recent years, the findings and experimental framework established in this study remain highly relevant and broadly applicable. The aged mouse model and multi-omics approach described here can be readily extended to other clinically important seasonal influenza viruses, including influenza B (Victoria lineage), A(H1N1)pdm09, and A(H3N2) strains. Such studies would further enhance our understanding of age-associated immune responses and disease pathogenesis across diverse influenza virus subtypes and support the development of improved vaccines and therapeutics for vulnerable elderly populations.

## Data Availability

We have submitted the RNA-seq data on GEO submission, and the submission ID is SUB16233714. We have included the biosample accession IDs of RNA-seq data in Table S2.

## SUPPLEMENTAL MATERIAL

Supplemental figures and tables spectrum.02656-25-s0001.docx Fig. S1 to S6, and Tables S1 and S2.

ASM does not own the copyrights to Supplemental Material that may be linked to, or accessed through, an article. The authors have granted ASM a non-exclusive, world-wide license to publish the Supplemental Material files. Please contact the corresponding author directly for reuse.
