# Supplementary material for: Systems-level insights into age-dependent innate immune responses in influenza B infection
Source: Microbiol Spectr. 2026 Aug 19;14(10):e02656-25. doi: 10.1128/spectrum.02656-25 (PMC13688880; doi:10.1128/spectrum.02656-25)
Supplement: Supplementary file 1 [file spectrum.02656-25-s0001.docx]

**Supplementary data**

**System-Level Insights into Age-Dependent Innate Immune Responses in Influenza B Infection**

**Authors:** Shikha Saxena^1#^, Gurleen Kaur^1,4#^, Preeti Vishwakarma^1^, Varun Kumar^1^, Amit Kumar^1^, Sonia Ompal^1^, Satish Kumar^1^, Sarjeet Singh^1^, Akanksha Chauhan^1^, Susmita Chaudhuri^2^, Waseem Akram Malla^3^, Sweety Samal^1^*

**Affiliations:**

^1^Center for Virus Research, Therapeutics and Vaccines, Translational Health Science & Technology Institute, NCR Biotech Science Cluster, Faridabad, Haryana, India-121001.

^2^Center for Microbial Research, Translational Health Science & Technology Institute, NCR Biotech Science Cluster, Faridabad, Haryana, India-121001

^3^ ICMR-National Institute of Malaria Research Field Unit Guwahati, Assam, India – 781036.

^#^Equal contribution

Running title: Age-Linked Innate Immunity in Influenza B

*To whom correspondence may be addressed.

**Dr. Sweety Samal***

Centre for Virus Research, Therapeutics and Vaccines,

Translational Health Science and Technology Institute, NCR Biotech Science Cluster, Faridabad, Haryana, 121001, India

E-mail: [sweety.samal@thsti.res.in](mailto:sweety.samal@thsti.res.in)

**Supplementary fig. 1-**

**Supplementary figure 1-** The growth kinetics of mouse adapted influenza B (Inf B/Phuket/3073/2013) in MDCK-L cells by Reed and Muench method. (A)The virus titer was done at 6h, 12h, 24h, 48h and 72h post infection. (B) The virus titer in aged BALB/c mouse in spleen, kidney, brain and liver at 3 day, 5 day and 7 day post challenge with their respective control. The experiment was done with (n=3) BALB/c mice for each time point and done in triplicates. Data are mean ± SEM (n=3) with p values <0.0001. The p value summary (*** p ≤ 0.001, ** p ≤ 0.01 and * p ≤ 0.05, n.s.: not significant).

**Supplementary fig. 2-**

**Supplementary figure 2**- (A& B) PCA plots for the Bulk RNA-seq data in the lung of influenza B/Phuket/3073/2013 infection at 5 days post challenge in young adult verses aged BALB/c mice control and influenza B infection

**Supplementary fig. 3-**

**Supplementary figure 3-** Reactome gene-concept network reveals enrichment of inflammatory and antiviral pathways in aged mice following Influenza B virus infection (A) The gene network for upregulated pathways (B) The gene network for downregulated pathways in aged mice following Influenza B virus infection

**Supplementary fig. 4-**

**Supplementary figure 4-** The proteomics analysis for the control adult and control aged female BALB/c mice (A) Venn diagram for the young adult and aged mice in which 578 differentially expressed protein were found to be common. (B) Heatmap for the differentially expressed proteins (DEPs) in lung at 5 days in young adult and aged mice (C) PCA plots for the proteomics data in young adult and aged mice (D &E) The Reactome pathways for upregulated and downregulated differentially expressed proteins (DEPs) in lung at 5 days in young adult and aged mice.

**Supplementary fig. 5-**

**Supplementary figure 5-** PCA plots for the proteomics data in the lung of B/Phuket/3073/2013 infection at 5 days post challenge (A) B/Phuket/3073/2013 infected young adult vs B/Phuket/3073/2013 infected aged BALB/c mice (B) Control young adult vs B/Phuket/3073/2013 infected young adult BALB/c mice (C) Control aged vs B/Phuket/3073/2013 infected aged lung of BALB/c mice.

**Supplementary fig. 6-**

**Supplementary figure 6-** Heat map for differentially expressed proteins (DEPs) in lung at 5 days post challenge in proteomics data (A) Influenza B/Phuket/3073/2013 infected young adult vs influenza B/Phuket/3073/2013 infected aged BALB/c mice. (B) Control young adult vs B/Phuket/3073/2013 infected young adult BALB/c mice (C) Control aged vs influenza B/Phuket/3073/2013 infected aged BALB/c mice

**Supplementary table- 1**: The primer sequences to evaluate the expression profile of the genes in adult and aged BALB/c mice in influenza B infection.

| Sl.no | Gene | Primer sequence |
| --- | --- | --- |
| 1 | Influenza B (M2) | For-CAAGACCAATCYTGTCACCTCTGAC  Rev-GCATTYTGGACAAAVCGTCTACG |
| 2 | Gapdh | For-CTCATGACCACAGTCCATGC  Rev-CACATTGGGGGTAGGAACAC |
| 3. | TLR-1 | For-CAATGTGGAAACAACGTGGA  Rev-TGTAACTTTGGGGGAAGCTG |
| 4. | TLR-2 | For-AAGAGGAAGCCCAAGAAAGC  Rev-CGATGGAATCGATGATGTTG |
| 5. | TLR-3 | For-CACAGGCTGAGCAGTTTGAA  Rev-TTTCGGCTTCTTTTGATGCT |
| 6. | TLR-4 | For-ACCTGGCTGGTTTACACGTC  Rev-CTGCCAGAGACATTGCAGAA |
| 7. | TLR-5 | For-AAGTTCCGGGGAATCTGTTT  Rev-GCATAGCCTGAGCCTGTTTC |
| 8. | TLR-6 | For-TTCCCAATACCACCGTTCTC  Rev-CTATGTGCTGGAGGGTCACA |
| 9. | TLR-7 | For-AATCCACAGGCTCACCCATA  Rev-CAGGTACCAAGGGATGTCCT |
| 10. | TLR-8 | For-GACATGGCCCCTAATTTCCT  Rev-GACCCAGAAGTCCTCATGGA |
| 11. | TLR-9 | For-ACTGAGCACCCCTGCTTCTA  Rev-AGATTAGTCAGCGGCAGGAA |
| 12. | ISG-15 | For-TGCCTGCAGTTCTGTACCAC  Rev-AGTGCTCCAGGACGGTCTTA |
| 13. | IFIT-2 | For-TCCTCCAAAAGGCAGCTCAG  Rev-TCCCTGTAACGGCACATGAC |
| 14. | IRF-7 | For-GTGACCCTCAACACCCTAATAC  Rev-CCACAGATAGTGTGACCAAGTT |
| 15. | IRF-9 | For-CTCCCAGTCCCAAACTCTAAATC  Rev-CAGTCTTCTTGCCTCTTGTCTC |
| 16. | IFN alpha | For-CAGTCCAGAGAGCCATCAATATC  Rev-TAGAGAGCAGGTTGACCAGTA |
| 17. | IFN Beta | For-GGAAAGATTGACGTGGGAGAT  Rev-CAGGCGTAGCTGTTGTACTT |
| 18. | IFN Gamma | For-AAAGAGATAATCTGGCTCTGC  Rev-GCTCTGAGACAATGAACGCT |
| 19. | STAT-1 | For- CTGGAGGAGTTGGAACAGAAA  Rev- CTGAATGAGCTGCTGGAAGA |
| 20. | IFIT-3 | For- CCTCAGAACCAGTACCTGAAAG  Rev- CTAATCAAACGGATGTCCTCCA |

**Supplementary table 2-** Bio sample accession ID of the RNA-seq data

| Sl. No. | Sample name | Accession ID |
| --- | --- | --- |
| 1 | Adult_Control_1 | SAMN60633710 |
| 2 | Controladultlung_2 | SAMN60633711 |
| 3 | Adult_Control_3 | SAMN60633709 |
| 4 | Adult_PhuketInfected_3 | SAMN60633712 |
| 5 | Phuketinfectedadultlung_1 | SAMN60633713 |
| 6 | Phuketinfectedadultlung_2 | SAMN60633714 |
| 7 | ControlAged_3 | SAMN60633715 |
| 8 | Control_Aged_4 | SAMN60633716 |
| 9 | Controlagedlung_1 | SAMN60633717 |
| 10 | Aged_PhuketInfected_3 | SAMN60633718 |
| 11 | Phuketinfectedagedlung_1 | SAMN60633719 |
| 12 | Phuketinfectedagedlung_2 | SAMN60633720 |
